# Graphitic Nitrogen and High‐Crystalline Triggered Strong Photoluminescence and Room‐Temperature Ferromagnetism in Carbonized Polymer Dots

**DOI:** 10.1002/advs.201801192

**Published:** 2018-11-13

**Authors:** Siyu Lu, Laizhi Sui, Min Wu, Shoujun Zhu, Xue Yong, Bai Yang

**Affiliations:** ^1^ State Key Laboratory of Supramolecular Structure and Materials College of Chemistry Jilin University Changchun 130012 China; ^2^ College of Chemistry and Molecular Engineering Zhengzhou University 100 Kexue Road Zhengzhou 450001 China; ^3^ Institute of Atomic and Molecular Physics Jilin University Changchun 130012 China; ^4^ College of Materials Science and Engineering Zhejiang University of Technology Hangzhou 310014 China

**Keywords:** carbonized polymer dots, graphitic nitrogen, high‐crystalline materials, photoluminescence, room‐temperature ferromagnetism

## Abstract

Carbonized polymer dots (CPDs) have great potential for bioimaging and biosensing owing to their low toxicity, low cost, resistance to photobleaching, and low environmental impact. Here, the hydrothermal condensation of biomolecules (l‐serine and l‐tryptophan) is used to vary the CPDs' inner structure from amorphous to lattice. A new type of carbon lattice CPD is thus demonstrated that is bright (the photoluminescence quantum yield (PLQY) is as high as 89.57%) and shows room‐temperature ferromagnetism (RTFM), with the magnetic moment increasing from 0.0025 emu g^−1^ in crosslinked polymer clusters to 0.021 emu g^−1^ in the latticed sample. Hydrothermal synthesis at 300 °C leads to a distinct type of CPD with an obvious carbon lattice, which shows the highest PLQY and the greatest ferromagnetism. Then, the origin of the RTFM is examined in the CPDs via first‐principles calculation, revealing that graphitic nitrogen triggers RTFM in CPDs. Moreover, a possible growth mechanism is suggested that includes kinetics as an important factor in the formation of the CPD crystallites. Overall, these findings identify graphitic nitrogen and high crystallinity as crucial to the enhancement of the CPDs' photoluminescence and room‐temperature ferromagnetism which suggests that they deserve more research attention to develop practical applications.

## 1 Introduction

In recent years, carbonized polymer dots (CPDs) comprising eco‐friendly carbon materials have emerged as fluorescent materials that fill the gap between semiconductor quantum dots and organic fluorophores. They have wide potential applicability in the fields of bioimaging, fluorescence sensors, and energy conversion due to their unique properties such as high water solubility, low toxicity, high optical and chemical stability, and flexibility of surface modification.^1^, ^2^ Various synthesis methods have led to CPDs with varying structures that show tunable properties that can fit many specific applications. CPDs with an amorphous carbon or polymer cluster structure always have a molecule‐based chromophore as the photoluminescence (PL) center, and show high PL quantum yield (PLQY).^3^ Those with an obvious carbon lattice have the whole carbon‐core state as the PL center. Nonradiative processes of the carbon core cause the PLQY of CPDs, and CPDs with a carbon lattice always show a lower PLQY than amorphous CPDs.^4^, ^5^, ^6^, ^7^

Ferromagnetism in carbon‐based materials is fundamentally and technologically important and has been sought for many years. Metal‐free carbon nanomaterials are promising candidates in, for example, spintronics, where carbon may easily integrate spin and molecular electronics into a single platform. The ferromagnetic properties of carbon‐based materials arise primarily due to (i) radicals that form chain‐like structures and interact with each other (long‐ and short‐range interactions); (ii) carbon mixtures comprising portions of sp^2^ and sp^3^ hybridized states; (iii) carbon structures incorporating impurities such as P, N, or B; and (iv) edge states (and defect sites). Each of these allow electron–electron interactions that cause magnetic polarization.

Control over CPDs' structure is a key objective to tune their properties for specific applications. As their synthesis reaction proceeds in a “bottom‐up” approach, these CPDs would go through further polymerization, dehydration, and carbonization.^8^, ^9^, ^10^ The composition of the CPDs' core can vary from amorphous to graphitic depending on the temperature of synthesis, with synthesis above 300 °C generally leading to significant graphitization, while amorphous particles result from cooler reactions, unless sp^2^/sp^3^ hybridized carbon has been included in the precursor. Therefore, CPDs obtained from “bottom‐up” approaches have either polymeric or polymer/carbon hybrid structures, and it is important to investigate and compare the PL of CPDs with and without a carbon lattice made using the same reaction system.^11^, ^12^, ^13^, ^14^, ^15^, ^16^ This work reports the preparation of a series of CPDs with systematic structure tuning via a uniform aminoacid reaction. With increasing synthesis temperature, the structure progressed from a crosslinked polymer cluster (sample CPDs100), via an amorphous carbon structure (CPDs200), to an obvious carbon lattice (CPDs300). It was surprising that the PLQY increased across the series from CPDs100 to CPDs300. CPDs with a perfect carbon lattice generally exhibit many nonradiative processes such as interlayer quenching. However, sample CPDs300 possessed a well‐developed carbon lattice, but showed the highest PLQY among the investigated CPDs. Obvious room‐temperature ferromagnetism was also observed in these carbon dots. As the carbon lattice and the presence of graphitic nitrogen increased in the series from CPDs100 to CPDs300, the magnetic moment increased from 0.0025 to 0.021 emu g^−1^.^17^, ^18^ It could open a new area of application for carbon dots and possibly lead to carbon‐based spintronic devices operating under ambient conditions.

## 2 Results and Discussion

**Figure 1:**
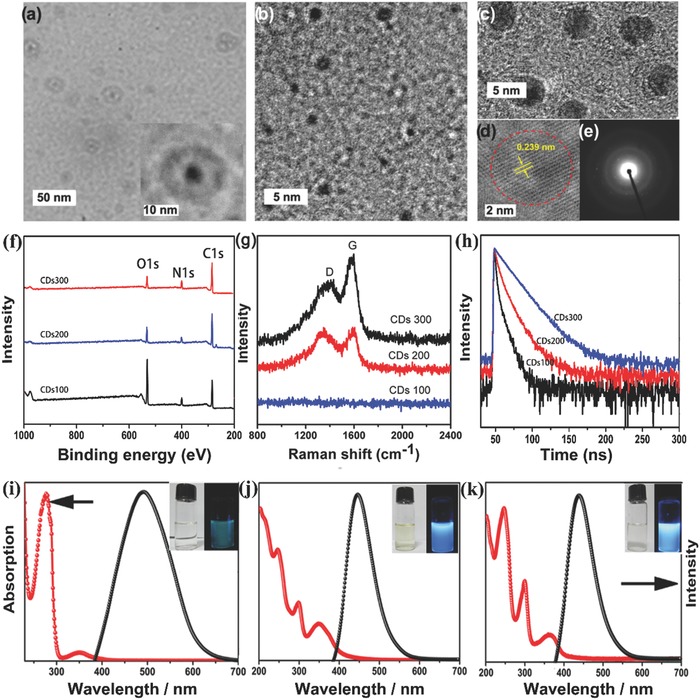
a) TEM and High‐Resolution TEM images (inset) of CPDs100; b) High‐Resolution TEM images of CPDs200; c–e) TEM, High‐Resolution TEM images, and electron diffraction images of CPDs300; f) XPS spectrums of CPDs300; g) Raman spectrums of CPDs100, CPDs200, and CPDs300; The time‐correlated single‐photon counting (TCSPC) of CPDs100, CPDs200, and CPDs300; h) PL spectra and UV–vis absorption of CPDs: i) CPDs100, j) CPDs200, and k) CPDs300 in aqueous solution.

The synthetic approach involved the heating of aqueous l‐serine and l‐tryptophan with acid (1 < pH < 3) at different temperatures. Heating at 100 °C for 8 h resulted in annular polymer dots (sample CPDs100) with an average diameter of 20 nm (the dots and the ring), which were detectable by transmission electron microscopy (TEM) (**Figure** **1**a). A hotter reaction (200 °C for 8 h) produced spherical CPDs (sample CPDs200) that possessed an amorphous carbon core structure (Figure 1b). They showed spherical symmetry and a relatively narrow size distribution, with an average diameter of about 2 nm. A yet hotter reaction (300 °C for 8 h) gave rise to clustering and the formation of larger and nonuniform carbogenic particles (sample CPDs300) with a mean diameter of 5 nm (Figure 1c). The in‐plane lattice spacing in CPDs300 (0.239 nm, red circle, Figure 1d) was very close to that of monolayer graphene, indicating a graphitic structure. X‐ray photoelectron spectroscopy (XPS) (Figure 1h) revealed three typical peaks for each sample, corresponding to C (285 eV), N (400 eV), and O (532 eV) (Figure 1f). The Raman spectra (Figure 1g) for CPDs200 and CPDs300 contain a G band at 1339 cm^−1^ and a D band at 1587 cm^−1^; CPDs100 showed no band. The G band corresponds to the E2g mode of graphite related to the vibration of sp^2^ hybrid carbon atoms, and the D band is ascribed to the vibrations of carbon atoms with dangling bonds in terminal planes of disordered graphite. The intensity ratio ID/IG therefore indicates disorder, and is 1.03 and 0.89 for CPDs200 and CPDs300, respectively, indicating increased crystallinity in the latter. Figure 1h shows long PL lifetimes on the order of nanoseconds over the whole emission spectrum of each of the three samples, indicating that they had only fluorescent properties. The variation in the lifetimes of CPDs100, CPDs200, and CPDs300 (4.3, 21.2, and 118.6 ns, respectively) indicates that the organic fluorophores were strongly affected by the surrounding environment. Overall, these characterizations indicate that CPDs300 possessed a highly ordered and crystalline structure.^15^, ^16^, ^17^

The optical properties of the three samples were investigated to reveal the effects of their different structures on their PL behaviors. Figure 1i–k shows their UV–vis spectra in aqueous solutions. CPDs100 showed a strong peak at around 276 nm and another absorption band at 350 nm. However, CPDs200 and CPDs300 showed peaks at 246 and 299 nm, with CPDs300 showing them more strongly than CPDs200. The peak at 350 nm shown by CPDs100 and CPDs200 was red shifted to 360 nm in the spectrum of CPDs300. This absorption peak change may be ascribed to increased graphitic N, which would cause electron enrichment. Both CPDs with graphitic cores (CPDs200 and CPDs300) showed significantly increased absorption at 300–800 nm (by 10 and 20 times, respectively) in comparison with the largely amorphous CPDs100. Moreover, the graphitized CPDs300 showed significantly enhanced light absorption compared with amorphous CPDs200, which displayed limited photosensitizer ability due to low extraction of photogenerated charges. The improved absorption of the CPDs is particularly significant at the tail into the visible wavelengths. This effect reflects an increased number of delocalized π–π* (C=C) optical transitions in the core as a result of the high sp^2^ carbon content of the graphitized CPDs and increased graphitic N.

The PL emission spectra show optimal excitation wavelengths of 325 and 388 nm for CPDs100, and 259, 302, and 372 nm for both CPDs200 and CPDs300. CPDs100 had their greatest emission at 489 nm, while the emission maxima for CPDs200 and CPDs300 were at similar wavelengths (437 and 434 nm, respectively). The largely similar absorption bands and emission peaks indicate that the PL in CPDs300 and CPDs200 arose mainly from the same fluorophores. Detailed PL studies of the three samples were carried out with different excitation wavelengths ranging from 300 to 420 nm. The PL of aqueous CPDs100 was excitation dependent. The optimal emission band was centered at λ_em_ = 489 nm, and showed maximum intensity at λ_ex_ = 380 nm. Longer excitation wavelength resulted in red‐shift of the emission wavelength to 505 nm. However, the emission from CPDs200 and CPDs300 was nearly excitation independent. CPDs100 appeared green under a UV lamp, while both CPDs200 and CPDs300 showed strong blue emission. CPDs300 exhibited the highest quantum yield and narrowest full width at half maximum (64 nm) of the three samples because of its high purity and perfect graphitization. PLQY data of these CPDs with greater accuracy were also gained through direct measurement using an absolute PLQY (internal quantum efficiency) measurement system. The PLQYs of CPDs100, CPDs200, and CPDs300 with 370 nm excitation were calculated to be 6.6%, 46.7%, and 89.57%, respectively. The PLQYs of CPDs300 was almost equivalent to the PLQYs of fluorescent dyes.^3^

**Figure 2:**
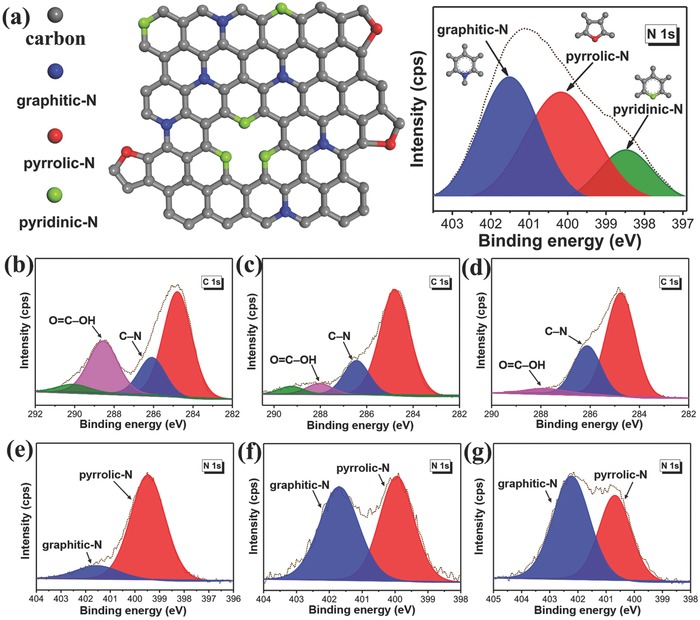
a) Scheme showing different bonding configurations of nitrogen in N‐doped graphene and corresponding peaks in a simulated high‐resolution N 1s XPS pattern. High‐resolution XPS C 1s and N 1s spectra of the b,e) CPDs100, c,f) CPDs200, and d,g) CPDs300.

**Figure** **2**a shows nitrogen in different configurations inside a graphene lattice or attached covalently to a graphene sheet, along with the three distinct peaks corresponding to each state. XPS analysis showed that the amount of oxygenous carbon strongly decreased as the reaction temperature increased (Figure 2b–d). Nitrogen originating from surface amide groups and pyrrolic‐N (399.7–400.1 eV) dominated in CPDs100 and CPDs200, whereas graphitic nitrogen was the prevailing structural motif in CPDs300 (Figure 2e–g).^19^ Hence, the high‐resolution N1s XPS spectra show that the most significant parameter responsible for the high PLQY and room‐temperature ferromagnetism (RTFM) was the increasing amount of graphitic nitrogen located around 401–402.3 eV. These findings are in a good agreement with the results mentioned above.

**Figure 3:**
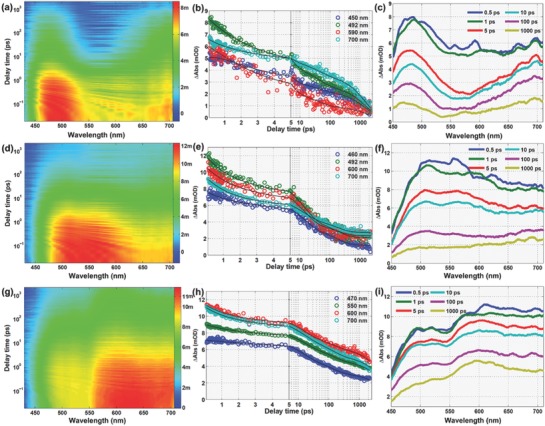
2D pseudocolor map of transient absorption (TA) spectra expressed in ΔOD as functions of both delay time and probe wavelength for the CNDS with pump wavelength of 400 nm, a) CPDs100, d) CPDs200, g) CPDs300; TA spectra of CPDs at indicated delay time from 0.5 ps to 1 ns, b) CPDs100, e) CPDs200, h) CPDs300; kinetic traces at different probe wavelength, black solid lines are global fitted curves, g) CPDs100, h) CPDs200, i) CPDs300.

Further analysis of the PL mechanism and the relaxation dynamics of excited hot carriers in the CPDs that formed at different temperatures was undertaken through femtosecond transient absorption (TA) spectroscopy using 400 nm excitation. **Figure** **3**a,d,g shows 2D transient absorption spectra expressed in Δ optical density (OD), as functions of both delay time (0 ps to 2 ns) and probe wavelength (450–700 nm). In all three TA spectra, there are only positive absorption change features (red), which should correspond to excited‐state absorption (ESA) in the whole probe range. The disappearance of negative signals, related to stimulated emission (SE) and ground‐state bleaching (GSB), is ascribed to the CPDs' high ESA in the visible range. The steady state absorption spectra of the CPDs are mostly located under 400 nm, thus excluding the influence of GSB on these results. Therefore, there are two superimposed signals, ESA and SE. Figure 3b,e,h presents the entire spectral evolution with different time delays and the kinetics of each featured wavelength in terms of the delay times for the various samples. Typical TA spectra (1 ps) of the CPDs are displayed in Figure 3c and Figure S1 in the Supporting Information. The ESA peaks of CPDs100 and CPDs200 are located at 480 and 520 nm, respectively. However, CPDs300 showed a broader and stronger band at 560–700 nm. The XPS results suggest that the differences in the TA spectra are strongly affected by the C—C/C=C ratio and functional groups containing nitrogen.

Four exponential decay functions were globally fitted to the TA data to explore the detailed relaxation channels of the excited carriers and the effect of the different carbon cores and surface states on the PL mechanism. The fitted lifetimes of carriers in CPDs100 are 0.63 ± 0.05 ps, 3.18 ± 0.04 ps, 39.1 ± 0.09 ps, and 1.48 ± 0.01 ns. Details of the changes in the spectra within different lifetime components can be gained from the decay associated difference spectra in Figure 3f and Figure S2a in the Supporting Information. The spectra are normalized to evaluate the proportion of each component in the entire carrier decay dynamics (Figure 3i; Figure S2e, Supporting Information). The second lifetime process accounted for the largest proportion, the spectral region of the first lifetime was distributed at 450–470 and 550–600 nm, and the last lifetime component was divided into two regions by 570 nm. The above analysis and results reported for carbon‐based materials using ultrafast technology led us to attribute four components to the corresponding relaxation channels. After 400 nm excitation, one part of the Coulomb‐induced hot carriers in the carbon core is trapped within 0.63 ps by surface states consisting of the carbon backbone and functional groups. The remaining hot carriers in the carbon core will release the redundant energy by optical photon scattering (3.18 ps) and acoustic photon scattering (39.1 ps). The surface states of CPDs100 are divided into two parts: nonluminous surface states that possess the same relaxation channels as the carriers in the carbon core, and luminous surface states that allow the recombination of electrons and holes within 1.48 ns.

The four fitted lifetimes of CPDs200 are 1.43 ± 0.01 ps, 14.6 ± 0.2 ps, 177 ± 3 ps, and 3.22 ± 0.05 ns. The disappearance of hundreds of femtoseconds in our fitted data is likely to have been related to the processes involving trapping by surface states being beyond our instrumental detection limit. The first and second lifetimes matched with optical and acoustic phonon scattering, and they become shorter with increasing synthesis temperature. Carriers in the amorphous carbon core structure and nonluminous surface states increased a new relaxation channel that provided transit to the ground state within 177 ps. However, the last lifetime related to fluorescence increased to 3.22 ns. The first three lifetimes (1.43 ± 0.01, 14.4 ± 0.2, and 124 ± 2 ps,) of CPDs300 are very similar to those of CPDs200, which demonstrates that the carriers in CPDs200 and CPDs300 experience the same relaxation channels. The luminous surface states emit fluorescence by the recombination of electrons and holes within the longest lifetime (7.23 ± 0.06 ns), which further explains why CPDs300 has the greatest PLQY. Transient absorption spectroscopy showed that nitrogen doping in CPDs increases the efficiency of hole scavenging by the electron donor and thereby significantly extends the lifetime of the photogenerated electrons.

The unexpected and important two‐photon fluorescence properties of the various CPDs were investigated using a near‐infrared (NIR) femtosecond pulsed laser (800 nm). A representative two‐photon luminescence spectrum of CPDs300 is shown in Figure S3a,c (Supporting Information) is similar to that for one‐photon fluorescence. However, two‐photon fluorescence only appeared in CPDs200 and CPDs300. The two‐photon luminescence under pulsed infrared laser excitation was confirmed by comparing different laser intensities for excitation. Figure S3b,d (Supporting Information) shows a clear linear relationship between the square of excitation laser intensity and the luminescence intensity, confirming that excitation with two NIR photons was responsible for the luminescence of the CPDs. When the excitation laser intensity and the OD of steady absorption at 400 nm were the same, the two‐photon PL intensity in the CPDs300 was nearly four times larger than that in CPDs200. The third‐order nonlinear susceptibility χ³ related to two‐photon absorption in CPDs was shown to be influenced by the nitrogen doping content and the carboxyl function groups through nanosecond and picosecond Z‐scanning.

**Figure 4:**
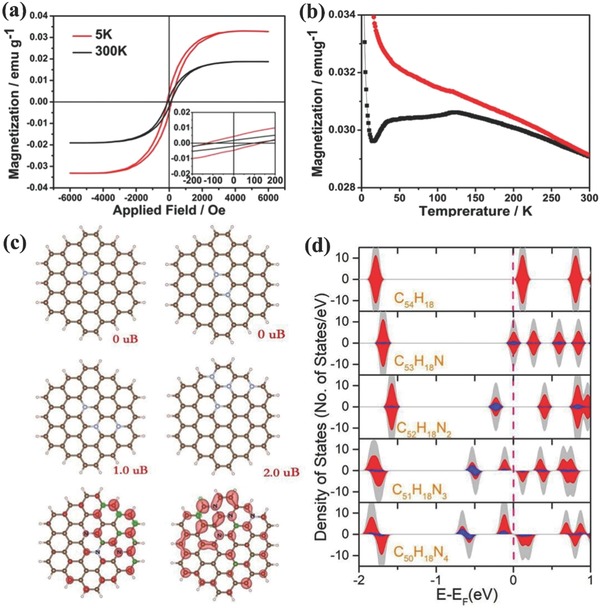
a) Field‐dependent magnetization (*M–H* curve) at 5K (red line) and 300 K (black line) for CPDs300, the insets shows the magnified views of the low‐field region; Origin of the magnetic properties in CPDs300.

The spin configurations of the three samples were subsequently detected using a superconducting quantum interference device magnetometer. Like most carbon materials, CPDs100 was nonmagnetic. CPDs200 possessed an amorphous structure with negligible ferromagnetism, as shown in Figure S4 in the Supporting Information. The field‐dependent hysteresis loop (*M*–*H*) curves of CPDs300 at 5 and 300 K are shown in **Figure** **4**a. The negligible remnant magnetization and coercivity in the hysteresis loops indicate room‐temperature ferromagnetism. Importantly, both precursor powders, l‐serine and l‐tryptophan, were measured to be nonmagnetic. Therefore, we conclude that the observed magnetism in CPDs300 is robust and an intrinsic property. Figure 4b depicts the temperature‐dependent magnetization of CPDs300 during field‐cooled (FC) and zero‐field‐cooled (ZFC) procedures under an applied field of 500 Oe. The ZFC magnetization increased gradually up to ≈170 K, and then declined slowly, suggesting that the blocking temperature (TB) was 170 K. This rise and fall behavior in the ZFC curve is understandable, because as the temperature increases to TB, the thermal energy disturbs the system and more moments acquire the energy to be aligned with the external field direction; above TB, the thermal energy is strong enough to randomize the magnetic moments, thus decreasing the magnetization. Moreover, the FC and ZFC plots exhibit distinct bifurcation at the onset near 300 K, which indicates the existence of magnetic ordering in the sample.

To explore the origin of the ferromagnetism in CPDs300, we simulated a series of possible models. The starting model for the study of N‐doping is a hypothetical graphene sheet modeled by a planar C_54_H_18_ flake. This model has been used in many previous theoretical studies. To investigate the magnetic properties of N‐doped graphene with different doping concentrations, structural models of C_53_H_18_N, C_52_H_18_N_2_, C_51_H_18_N_3_, and C_50_H_18_N_4_ were employed (Figure 4c) with N concentrations of 1.9%, 3.7%, 5.6%, and 7.4%, respectively. A large supercell of size 25 Å × 25 Å × 15 Å was used to simulate a separate graphene flake without inter‐molecular interactions due to the periodic boundary conditions. During the geometry optimization, the cell was fixed and all the atoms were allowed to optimize. The first‐principles calculations show that the pristine graphene flake C_54_H_18_ without N‐doping is an insulator with no magnetic moment. The N atom has one more electron than the C atoms, indicating that it acts as a charge donor when doped into a graphene flake replacing one C atom. Doping graphene flakes with one or two N atoms did not lead to a magnetic moment. However, further N doping induced magnetic moments of 1.0 and 2.0 µB in the C_51_H_18_N_3_ and C_50_H_18_N_4_ structures, respectively. This is consistent with previous findings of no magnetic moment emerging until the N doping concentration is above 5%. Figure 4c shows the spin charge distributions of C_51_H_18_N_3_ and C_50_H_18_N_4_. The spin charges were not localized around the doped N atoms. The spin‐polarized electronic densities of states (DOS) of all the studied models are shown in Figure 4d. Spin‐polarization can be clearly seen in the projected density of states (PDOS) of the C_51_H_18_N_3_ and C_50_H_18_N_4_ structures. Taking the Fermi level as a reference, the conduction bands were shifted down upon N doping, given that the doped N atoms were charge donors (as mentioned above). This is the first report of graphitic nitrogen triggering room‐temperature ferromagnetism in CPDs. Hence, this work opens an important new area of applicability for CPDs.

The CPDs formed by nucleation and growth, and assumed different structures depending on the temperature maintained during growth (100, 200, or 300 °C). The initial condensation of l‐serine and l‐tryptophan formed peptide chains and polymer dots at 100 °C. This simple intermolecular condensation reaction was accompanied by a loss of water molecules, leading to CPDs100 precursor with low quantum yield and no ferromagnetism. The PL spectrum was mostly due to oxygen‐containing fluorophores (green groups) and crosslink‐enhanced emission. Synthesis at 200 °C carbonized the peptide chains and polymer dots to form carbon cores with accelerated dehydration, which induced volume shrinkage. As a result, CPDs200 was smaller than CPDs100. Moreover, in this process, molecular fluorophores containing many more amide groups (blue groups) were formed. Therefore, the PL of CPDs200 mostly or exclusively arose from lattice‐free carbogenic cores and surface molecule state emission, which exhibited a strong blue emission and low RTFM. The transition from CPDs200 to CPDs300 follows behavior similar to Ostwald's step rule. The carbon core that formed at a lower temperature (≈200 °C) lacked a crystal lattice, and CPDs200 did not form a more‐stable crystal structure possessing the lowest free energy. However, formation at 300 °C caused the lattice‐free carbon core to be unstable due to increased interfacial energy, and it dissolved gradually into solution. It then reformed as large crystallites of kinetically stable CPDs300 with a clear crystal lattice. Overall, this suggests that kinetics played an important role in the formation of the CPDs300 crystallites. During the growth of CPDs300, the graphitic N‐containing fluorophores were locked into the stable crystal structure, and their nonradiative processes (vibration, rotation, etc.) were severely limited. As the carbon dots grew, the CPDs connected the organic molecules and inorganic nanocrystals. The postulated mechanism can apply to almost all CPDs formed via bottom‐up syntheses.^16^, ^18^

## 3 Conclusion

In summary, crosslinked polymer clusters, an amorphous carbon structure, and an obvious carbon lattice were achieved by altering the temperature in one synthesis system. The relationships between the structure and the corresponding fluorescence and ferromagnetism were then investigated. As the presence of graphitic nitrogen and the level of crystallization increased, the CPDs' PL and RTFM were strongly enhanced: the PLQY reached a maximum of 89.57%, and the magnetic moment increased from 0.0025 to 0.021 emu g^−1^. We next explored the origin of RTFM via first‐principles calculations. Strong RTFM was induced when N was doped at a concentration exceeding 5%. Graphitic N containing fluorophores and the highly crystalline structure played the most important roles in enabling strong PL and RTFM. We further suggested a possible growth mechanism of the CPDs, which indicates that kinetics was important in the formation of the CPDs300 crystallites. Further studies exploring the relationship between PL and ferromagnetism are underway in our laboratory.

## Conflict of Interest

The authors declare no conflict of interest.

## Supporting information

Supplementary
