# Supplementary material for: Graphitic Nitrogen and High‐Crystalline Triggered Strong Photoluminescence and Room‐Temperature Ferromagnetism in Carbonized Polymer Dots
Source: Adv Sci (Weinh). 2018 Nov 13;6(2):1801192. doi: 10.1002/advs.201801192 (PMC6343063; doi:10.1002/advs.201801192)
Supplement: Supplementary file 1 — Supplementary [file ADVS-6-1801192-s001.pdf]

Copyright WILEY-VCH Verlag GmbH & Co. KGaA, 69469 Weinheim, Germany, 2018.

ADVANCED SCIENCE
Open Access

Supporting Information

for *Adv. Sci.,* DOI: 10.1002/advs.201801192

Graphitic Nitrogen and High-Crystalline Triggered Strong Photoluminescence and Room-Temperature Ferromagnetism in Carbonized Polymer Dots

*Siyu Lu, Laizhi Sui, Min Wu, Shoujun Zhu, Xue Yong, and Bai Yang**

Supporting Information

Graphitic Nitrogen and High-crystalline Triggered Strong Photoluminescence and Room-temperature Ferromagnetism in Carbonized Polymer Dots

*Siyu Lu, Laizhi Sui, Min Wu, Shoujun Zhu, Xue Yong and Bai Yang**

Dr. S. Lu, Dr. S. Zhu, Prof. Dr. B. Yang

State Key Laboratory of Supramolecular Structure and Materials College of Chemistry, Jilin University

Changchun 130012 (China) E-mail: byangchem@jlu.edu.cn

Dr. S. Lu, Dr. X. Yong

College of Chemistry and Molecular Engineering Zhengzhou University 100 Kexue Road, Zhengzhou 450001 (China)

Dr. L. Sui

Institute of Atomic and Molecular Physics, Jilin University Changchun 130012 (China)

Dr. M. Wu

College of Materials Science and Engineering Zhejiang University of Technology Hangzhou 310014, China

Experimental section

Preparation of Carbonized Polymer Dots: The typical experimental procedure is described as follows: L-serine (0.157 g) and L-tryptophan (0.1022 g) were dissolved in 10 ml weak alkaline water (about pH ～ 4) adjusted by adding HCL. Then the mixture was transferred to a poly(tetrafluoroethylene) (Teflon)-lined autoclave (30 ml) and heated at 100**,** 200 and 300°C for 8 h. The reactor was automatically cooled to room temperature. After this reaction, a clear golden yellow solution was obtained. Remarkably, the as-synthesized sample is found to exhibit favorable aqueous dispersion. The reactor was automatically cooled to room temperature. Then, the reactor was soaked by enthanol and the enthanol solution was centrifuged at 16 000 r min.1 for 10 min twice to wash off impurities.

Characterization: High-resolution transmission electron microscopy (HTEM) was performed on an FEI Tecnai F20. TEM was conducted using a Hitachi H-800 electron microscope at an acceleration voltage of 200 kV with a CCD cinema. Fluorescence spectroscopy was performed using a Shimadzu RF-5301 PC spectrophotometer. UV-vis absorption spectra were obtained using a Shimadzu 3100 UV-vis spectrophotometer. IR spectra were recorded on a Nicolet AVATAR 360 FT-IR spectrophotometer. The confocal microscopy images were recorded using an Olympus Fluoview FV1000. X-ray Photoelectron Spectroscopy (XPS) was performed using an ESCALAB 250 spectrometer with a mono X-ray source Al Kα excitation (1486.6 eV). Binding energy calibration was based on C 1s at 284.6 eV.

Femtosecond transient absorption setup: A regeneratively amplified Ti:sapphire laser system (Coherent Libra, 50fs, 1kHz) provides the fundamental light source. The pump pulse (400nm) is generated by focusing a portion of fundamental light into BBO crystal. In order to avoid the influence of rotational relaxation effects on dynamics, the polarization of pump pulse is randomized by depolarizing plate. The other fundamental pulse provides broadband probe pulse (white light continuum) that is produced by focusing 800 nm fundamental light into sapphire plate (3mm). The pump and probe beams are overlapped in the sample with crossing areas of 600 μm and 150 μm. After passing through the sample, the probe pulse is focused into optical fiber that is coupled to spectrometer(AvaSpec-1650F). The energy of 400 nm excitation pulse is adjusted to about 1.5μJ/pulse by a neutral density optical filter. The

pump pulse is chopped at 500 Hz to acquire pumped (signal) and un-pumped (reference) probe spectra, and the ΔOD spectrum can be obtained by processing them. The solutions are placed in 2 mm optical path length quartz cuvette. Both the instrument response function (100 fs) and temporal chirp in the probe light are determined by measuring the cross modulation of ethanol. The group velocity dispersion effect on the experiment data is corrected by home-made chirp program. For each measurement, the pump-probe delay scan is repeated three times to give the averaged experiment data.

Two-photon fluorescence (TPF) setup: The excited source consists of a regeneratively amplified Ti:sapphire laser system (Coherent Libra, 50fs, 1kHz, 800 nm). The TPF signal is collected at the right angles from excited solution placed in the 1cm fluorescence cuvette .The signal is coupled into the spectrometer (Spectra Pro 500i,PI Acton) through a fiber. Finally, the TPF signal is detected by an intensified charge coupled device (ICCD, PIMAX4, Princeton Instruments) triggered by laser.

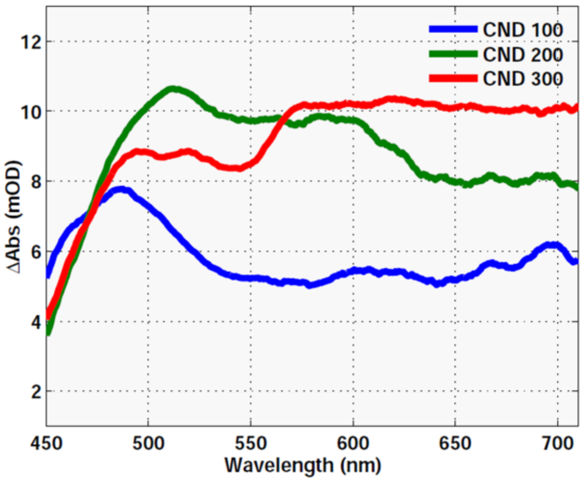

Figure S1 Typical transient absorption spectrum (at 1 ps) for three CNDs with different synthesis temperature at 400-nmexcitation.

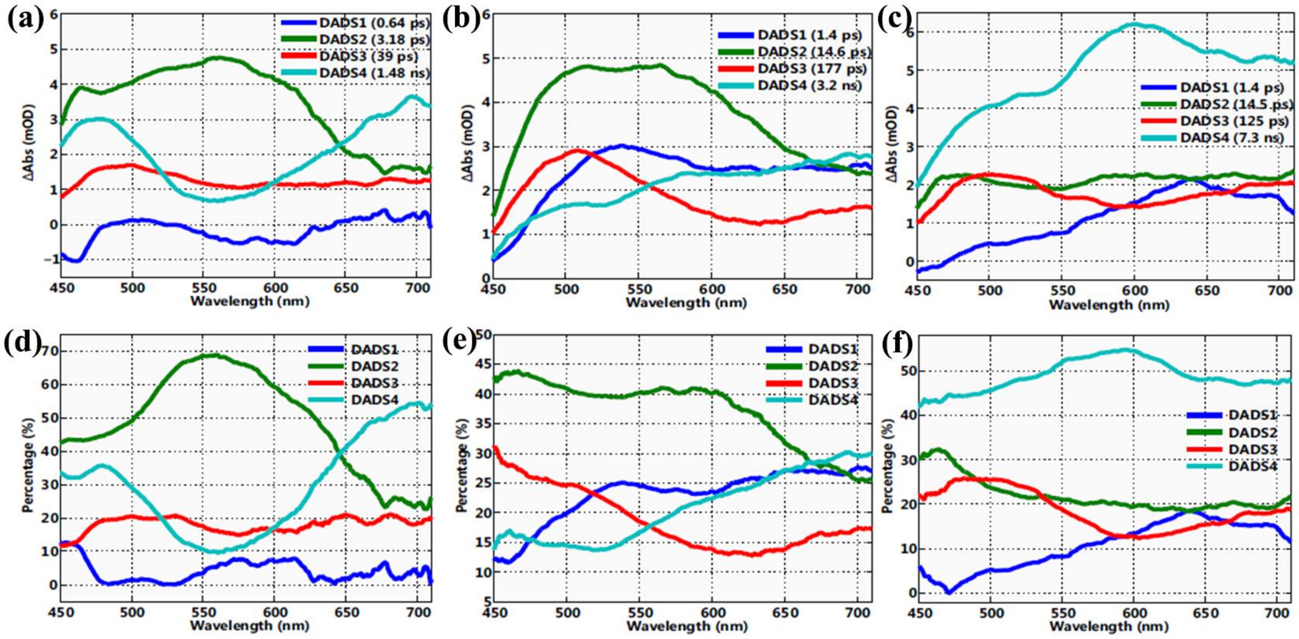

Figure S2 Results of the global fitting with four exponent decay functions showing four decay associated difference spectra (DADS), (a) CDs100 (b) CDs200 (c) CDs300; The percentage of the four decay processes occupied in the total dynamic within different wavelength according to DADS, (d) CDs100 (e) CDs200 (f) CDs300.

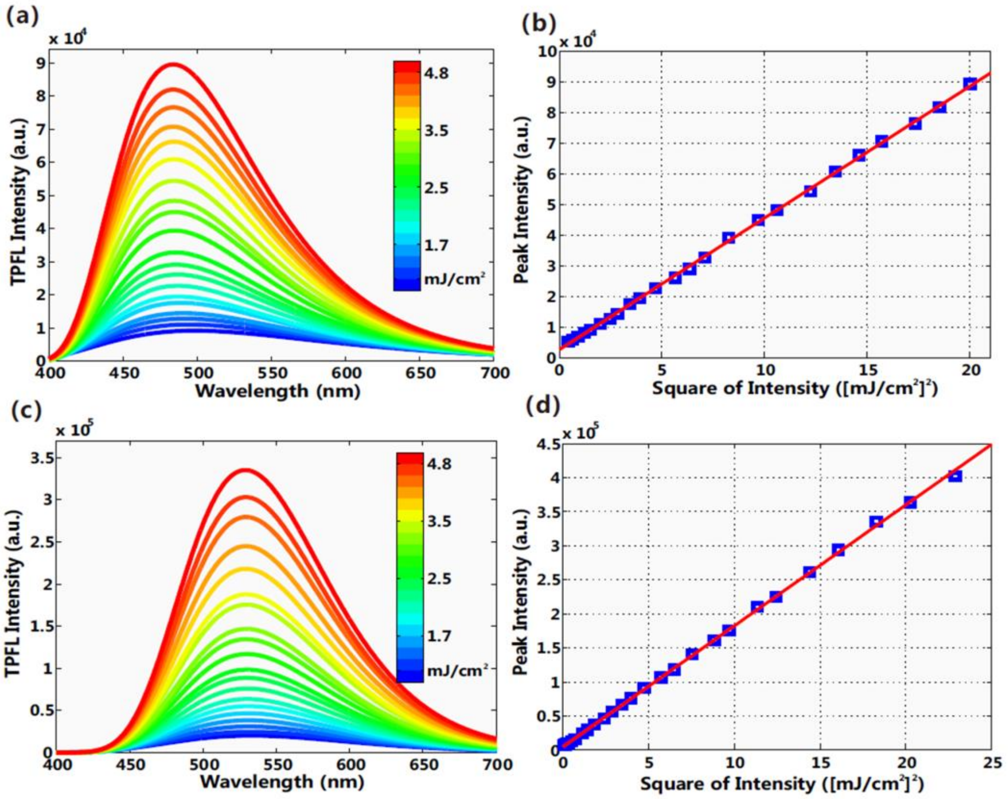

Figure S3 The two-photon PL spectra with different laser excitation intensity of 800 nm femtosecond pulse laser,(a) CDs 200 (c) CDs300; the relationship between the two-photon emission intensity and square of laser excitation intensity, (b) CDs 200 (d) CDs300.

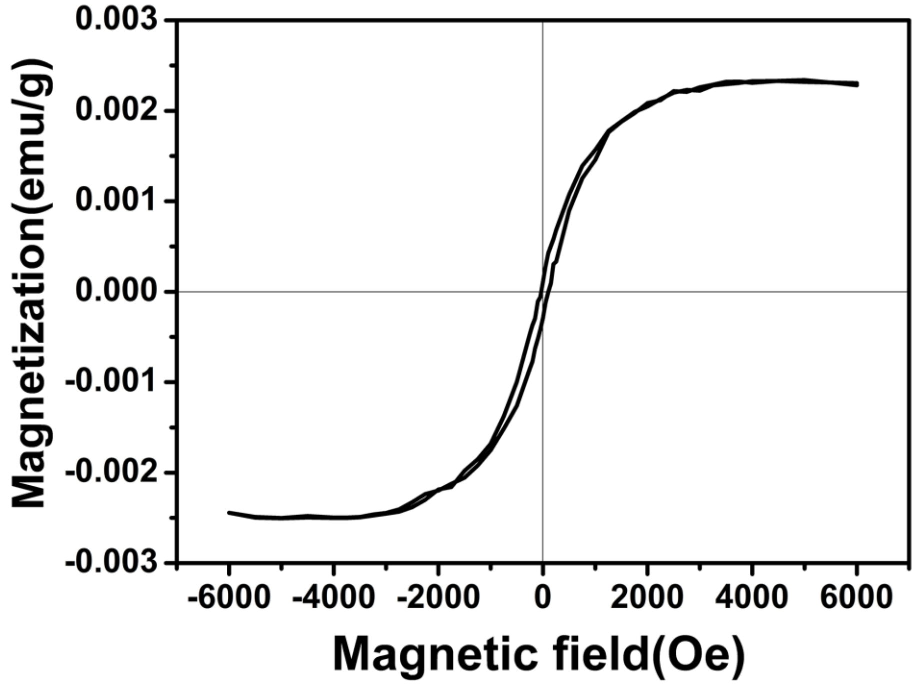

Figure S4 Field-dependent magnetization (M-H curve) at 300 K for CDs200
